# Multiple organ dysfunction - we must take a closer look at muscle wasting!

**DOI:** 10.5935/0103-507X.20190021

**Published:** 2019

**Authors:** Cassiano Teixeira, Felippe Leopoldo Dexheimer Neto, Régis Goulart Rosa

**Affiliations:** Intensive Care Unit, Moinhos de Vento Hospital - Porto Alegre (RS), Brazil; Internal Medicine, Universidade Federal de Ciências da Saúde de Porto Alegre - Porto Alegre (RS), Brazil.; Intensive Care Unit, Hospital Ernesto Dornelles - Porto Alegre (RS), Brazil.; Intensive Care Unit, Moinhos de Vento Hospital - Porto Alegre (RS), Brazil.

**To the editor**

Organ dysfunction is quantified routinely by scores such as: the Sequential Organ Failure Assessment (SOFA) score, the Multiple Organ Dysfunction Score (MODS), and the Logistic Organ Dysfunction Score (LODS).^(1)^ Once the number of organs failure is associated with intensive care unit (ICU) and hospital-mortality, these scores are helpful to only predict acute mortality.^(1)^ Our concern is that these tools focus exclusively on the same six organ systems - circulatory, renal, pulmonary, gastrointestinal and hepatic, hematologic, and central nervous systems. Is it enough?

As an organ dysfunction, muscle wasting perceived as ICU acquired weakness, is independently associated with short- and long-term morbidity and mortality.^(2)^ Just a few days of critical illness leads to significant amounts of lean body mass loses despite optimal nutrition, causing profound weakness (catabolism), recurrent nosocomial infections (immunosuppression), poor wound healing, and sepsis recidivism.^(3)^ During acute illness, muscle protein breakdown is permanently elevated with the pattern of intracellular signaling supporting increased breakdown and decreased synthesis, leading to a macro and microcirculatory dysfunctions.^(2,3)^ These pathologic processes participate in short-term failure of vital organs immediately threatens patient survival, and long-term recovery that is also severely hindered by persistent dysfunction of skeletal muscle and peripheral blood mononuclear cells.^(3)^ Further, ICU-acquired weakness is clinical consequence of skeletal muscle mitochondrial dysfunction, which occurs simultaneously in respiratory and locomotive muscles.^(2)^

In long-term follow-up, respiratory and neurologic systems are the only organic dysfunctions associated with mortality after ICU-discharge,^(3)^ however prolonged-mechanical ventilation dependence would not be a proxy for muscle weakness? Intensive care unit-acquired weakness worsens the prognostic of patients during ICU-stay,^(3)^ and its persistence and severity a great predictor of post-ICU mortality.^(4)^ Besides that, the recovery of the muscle power after hospital discharge may not reduce this risk.^(5)^ In addition, muscular weakness and functional disability can persist as long as five years after critical illness.^(5)^

In summary, the real importance of the concept of multiple organ failure in critical care setting is to predict acute and long-term mortality. Muscle dysfunction, i.e. acute muscle weakness, seems to be the major long-term predictor of mortality and must be included in scores of multiple organ failure.

*Cassiano Teixeira* *Intensive Care Unit, Moinhos de
Vento Hospital - Porto Alegre (RS), Brazil. Internal Medicine, Universidade
Federal de Ciências da Saúde de Porto Alegre - Porto Alegre (RS),
Brazil.* *Felippe Leopoldo Dexheimer
Neto* *Intensive Care Unit, Hospital Ernesto Dornelles -
Porto Alegre (RS), Brazil.* *Régis Rosa Goulart* *Intensive Care Unit,
Moinhos de Vento Hospital - Porto Alegre (RS), Brazil.*
